# Development of the Diabetes Indicators and Data Sources Internet Tool (DIDIT)

**Published:** 2005-12-15

**Authors:** Qaiser Mukhtar, Prachi Mehta, Erica R Brody, Jenny Camponeschi, Michael Friedrichs, Brenda Ralls, Angela M Kemple

**Affiliations:** Division of Diabetes Translation, Centers for Disease Control and Prevention; Northrop Grumman Mission Systems, Atlanta, Ga; RTI International, Research Triangle Park, NC; Wisconsin Department of Health and Family Services, Division of Public Health Diabetes Prevention and Control Program, Madison, Wis; Diabetes Prevention and Control Program, Utah Bureau of Health Promotion, Utah Department of Health, Salt Lake City, Utah; Diabetes Prevention and Control Program, Utah Bureau of Health Promotion, Utah Department of Health, Salt Lake City, Utah; Oregon Department of Human Services, Diabetes Prevention and Control Program, Portland, Ore

## Abstract

Developing a Web-based tool that involves the input, buy-in, and collaboration of multiple stakeholders and contractors is a complex process. Several elements facilitated the development of the Web-based Diabetes Indicators and Data Sources Internet Tool (DIDIT). The DIDIT is designed to enhance the ability of staff within the state-based Diabetes Prevention and Control Programs (DPCPs) and the Centers for Disease Control and Prevention (CDC) to perform diabetes surveillance. It contains information on 38 diabetes indicators (measures of health or factors associated with health) and 12 national- and state-level data sources. Developing the DIDIT required one contractor to conduct research on content for diabetes indicators and data sources and another contractor to develop the Web-based application to house and manage the information. During 3 years, a work group composed of representatives from the DPCPs and the Division of Diabetes Translation (DDT) at the CDC guided the development process by 1) gathering information on and communicating the needs of users and their vision for the DIDIT, 2) reviewing and approving content, and 3) providing input into the design and system functions. Strong leadership and vision of the project lead, clear communication and collaboration among all team members, and a commitment from the management of the DDT were essential elements in developing and implementing the DIDIT. Expertise in diabetes surveillance and software development, enthusiasm, and dedication were also instrumental in developing the DIDIT.

## Introduction

The Diabetes Indicators and Data Sources Internet Tool (DIDIT) is a Web-based resource designed to strengthen the capacity of the staff and partners of state-based Diabetes Prevention and Control Programs (DPCPs) and staff of the Centers for Disease Control and Prevention (CDC) to conduct diabetes surveillance and program evaluation. The tool contains detailed information on 38 diabetes indicators (measures of health or factors associated with health) and their associated data sources (e.g., Behavioral Risk Factor Surveillance System [BRFSS]). The content, design, and function of the DIDIT have been described elsewhere (1). This article describes the process of developing the DIDIT, beginning with the conceptual phase and proceeding through the content and systems phases (Table 1). In so doing, we provide an example for other agencies and organizations as well as other entities within the CDC that are interested in developing a similar tool.

## Developing the Concept and Content

The DIDIT was developed in response to a request from the DPCPs for technical assistance in surveillance and program evaluation (2). In August 2001, a work group comprised of representatives from the CDC’s Division of Diabetes Translation (DDT) and eight DPCPs (representing Alabama, Minnesota, Montana, New Mexico, New York, Oregon, Utah, and Wisconsin) was convened to develop a tool that would provide comprehensive information about 1) diabetes indicators (e.g., foot examinations, diabetes-related hospitalizations) and 2) their associated data sources (e.g., BRFSS, Medicare) in one centralized place. The work group generated a list of 55 indicators (later reduced to 38) and developed an outline describing the desired contents and format of a tool called the *diabetes indicator tool*.

In October 2001, an overview and vision of the DIDIT was presented to six focus groups at the annual meeting of DPCP directors. Three key themes emerged: 

- The DIDIT should be a Web-based application to allow for content updates and easy accessibility (in contrast to a CD-ROM).
- The DIDIT should be a reference tool that promotes consistency and standardization of data analysis required for diabetes surveillance.
- The tool's development should continue to be informed by DPCP representatives (the intended user group) to ensure that it meets the needs of program staff involved in diabetes surveillance.

Input from these initial focus groups and subsequent feedback from the DPCPs served as the basis for developing the concept, content, and Web application for the DIDIT.

### Development of content

Content development took place in two stages. During the first stage, the work group selected 10 of the originally identified 55 indicators to develop a prototype. The 10 indicators were as follows: 1) diabetes prevalence, 2) annual hemoglobin A1c test, 3) annual influenza vaccination, 4) pneumococcal vaccination, 5) level of diabetes education, 6) diabetes-related hospitalizations, 7) prevalence of end-stage renal disease, 8) hospitalization for lower extremity amputations, 9) physical inactivity, and 10) overweight. Because members of the work group lived in different states, discussions were conducted through a series of telephone conferences and two in-person meetings.

During the second stage, the work group selected an additional 28 indicators from the original 55 through a two-round modified Delphi process. Indicators were ranked in priority according to the following four criteria:

- Relationship to a national policy objective (such as the DDT's national diabetes objectives [2] or *Healthy People 2010* objectives [3])
- Alignment with current practice guidelines, such as those from the American Diabetes Association (4) 
- Responsiveness to efforts of the DPCPs
- Measurability through public data sources, particularly state-level data such as the BRFSS (5)

The typical reason for excluding an indicator was that no state-level data source could be identified to measure it. A list of indicators that were excluded and the rationale for excluding them can be found on the DIDIT (available from www.cdc.gov/diabetes/statistics/index.htm). All 10 indicators used to develop the prototype as well as all 28 selected during the second stage were retained, with a total of 38 indicators selected for inclusion. At this stage, the selection of fields to describe each indicator (e.g., definitions of indicators) and data source (e.g., its methodology for data collection, data access) was also finalized with input from the DIDIT work group.

After selecting indicators, associated data sources, and their related fields of information, the DIDIT team, including work group members, contractors for both content and Web site development, and DDT leaders, met in January 2003 (Table 1). The major focus of the meeting was to develop a process to ensure that the information included in the DIDIT was accurate and complete. The work group viewed the Web-based prototype with 10 indicators and eight associated data sources. Each work group member was assigned a set of indicators from the group of 38 to review and revise.

During content development, a contractor researched information on selected indicators and their associated data sources, and the work group provided feedback on its accuracy and completeness. Comments and suggestions on content were discussed during monthly conference calls, and the content was approved by all work group members before it was finalized. A second in-person meeting was held to review and refine content and to ensure that the process of reviewing content was efficient and effective. The protocol for developing and refining content resulted in valuable end-user feedback. Development of the Web-based application took place at the same time as development of content.

## Developing the Web-based Application

The parallel development of the application and its content relied on the software development life cycle (Table 2). This process included the following four major phases: 1) planning, 2) analysis, 3) design, and 4) implementation (6). We adopted an iterative and incremental approach, with overlap between analysis, design, and implementation.

### Phase 1: planning

The primary purpose of *project planning* was to articulate the objectives and scope of the DIDIT and ensure the technical feasibility of the system. We noted previously that the purpose and vision for the DIDIT were defined early during the concept development phase. During the project planning phase, the work group articulated the purpose and vision to the technical contractors, and they produced a document that outlined the definition and scope of the project; this document served as a blueprint for system development efforts. Additional activities in this phase included confirming the project's operational and organizational feasibility, developing a schedule, estimating costs, and allocating resources. The project planning phase culminated in the development of a final project plan, which was reviewed by the project lead and management staff. After the plan was approved, technical development efforts, or *systems analysis*, for the DIDIT were initiated.

### Phase 2: analysis

The *analysis* phase defined in detail what the information system needed to accomplish to provide users with the benefits they desired. Several storyboards were created to display preliminary design options for Web page content and format. The storyboards were uploaded onto a secure Internet site to allow sharing among and feedback from a geographically dispersed user group. As design options were presented, users were quickly able to provide comments. An iterative feedback process allowed further revisions to DIDIT storyboards. System requirements were prioritized as they were identified. The analysis phase culminated with the development of model diagrams, which were used to drive the next phase, *system design*.

### Phase 3: design

While the *analysis* phase focused on what the system should do, the *design* phase focused on how the system should function. Information from the analysis phase was used to design the application, the database, the user interface, and the operating environment. The application and database were designed in parallel with the user interface. The user interface is a critical component because it ensures ease of use. It was designed with stakeholder input to ensure that the final product would reflect stakeholder needs.

### Phase 4: implementation

During the *implementation* phase, a demonstration model was built, tested, and released with information on the 10 pilot indicators and associated data sources. The model included core functionalities such as the ability to browse, sort, and search, and it was demonstrated at the first in-person work group meeting in early 2003. Input was solicited on additional features, including the addition of DPCP-specific data sources, system-searching functions, and report formats.

#### Phase 4a: Merging the content and the Web system

After the demonstration model was developed, the development of the content and the Web system converged. The work group began using the DIDIT to review and refine the content of indicators and data sources. The contractor responsible for researching and developing the content delivered a data set on indicators and data sources to the systems developers for upload into the DIDIT. Once uploaded, the content was reviewed by the work group, and appropriate modifications were made. This process took place iteratively between February and April 2003, with a total of three uploads, until all 38 indicators and 12 data sources had been uploaded, reviewed, and finalized. An unexpected positive outcome from this process was that as the work group reviewed DIDIT content, it also tested and evaluated system functionality and design, leading to several important changes.

**Table:** 

**Rationale for Conducting Pilot Tests of the Diabetes Indicators and Data Sources Internet Tool (DIDIT)**
1. Provides validation that system function, design, and content are consistent with the responses elicited from users during the processes of requirements gathering and usability testing. This validation closes the information loop and confirms earlier assumptions.
2. Enables exploration of requirements or ideas suggested by users after the processes of requirements gathering and usability testing are complete. Although it might be too late to include these features in the first release, they can be incorporated into later phases.
3. Allows users to work with a real-life model, permitting them to visualize and respond to more advanced requirements they may find difficult to comprehend without such a model. Users also understand more advanced requirements when they can work with a system designed for fundamental needs and functions. In addition, requirements often build on one another.
4. Permits testing among a small subset of a large population of users, preferably subsets that differ from those selected in earlier development phases. This ensures a more representative sampling throughout the development process, and it ensures that feedback is well-rounded and unbiased. Although not all suggestions made during the pilot-testing phase are ultimately incorporated, the process often sparks ideas for future enhancements and provides insights for training and user support.

Before the DIDIT was formally implemented in September 2003, both usability testing and pilot testing were conducted to obtain user feedback about system design and usage to further refine the new tool. Usability testing was conducted in April 2003 at the DDT national conference, with pilot testing in July and August 2003 through conference calls and NetMeeting (Microsoft Corp, Redmond, Wash). NetMeeting allowed participants throughout the nation to view the DIDIT as it was being demonstrated at the CDC in Atlanta, Ga. Pilot testing is a critical and often overlooked component of the software life cycle, and there are important reasons for conducting it (Sidebar). The objective of usability testing and pilot testing was to formally validate that system function, design, and content were consistent with the needs of users as determined during earlier phases. Feedback from these processes was evaluated and used to make further refinements to the system before its final release in September 2003.

#### Phase 4b: System maintenance and training

Shortly after the release of the DIDIT, the project lead conducted a national training session for DPCPs and CDC staff using NetMeeting. A team of DDT professionals was then assigned the responsibility of providing ongoing user support and training for technical and functional aspects of the DIDIT. The project lead's responsibilities included providing support on questions and issues related to the content and application of the DIDIT in the context of DPCP programs.

## Elements That Facilitated the Development of the DIDIT

Several factors were critical to successfully developing and implementing the DIDIT. The factors have practical implications for other agencies that want to undertake a similar effort.

The work group members had extensive knowledge and experience in diabetes surveillance and epidemiology, which proved essential in guiding the content and technical contractors during the development process. DIDIT team members were a motivated, dedicated, enthusiastic, and knowledgeable group of DPCP representatives and DDT staff. In addition, the knowledge and skills of the contractor were critical to researching and developing content on indicators and data sources.

### Buy-in of management

The project lead effectively solicited the interest and support of DDT management to ensure that financial and staff resources were available to develop the new tool. To sustain interest and support of management, the project lead presented draft content and DIDIT prototypes at various CDC and national public health meetings throughout the development process (Table 1). These presentations allowed management to realize the high level of interest among prospective users and the potential for the DIDIT as an important tool for diabetes surveillance. Updates were also shared with DDT management on an ongoing basis.

### Commitment of time and resources

Development of a comprehensive reference tool such as the DIDIT requires a commitment of time and resources. The management of the DDT supported allocation of resources and time needed to create the DIDIT.

### Strong leadership and clear vision

The DIDIT project lead had a clear vision of the type of tool that would fulfill the surveillance needs of the DPCP and the DDT. A strength of the project lead was her ability to communicate the vision of the DIDIT to the project team and stakeholders throughout the development process.

### Collaboration among stakeholders and contractors

Development of the DIDIT involved input from stakeholders across the country. Clear and ongoing communication among stakeholders was essential to the development process. During the first in-person work group meeting, we learned that face-to-face interactions were highly appreciated by work group members and that these interactions helped build rapport among members. In-person meetings were arranged at national conferences to avoid issues of travel approval and costs. Timelines and other defined plans facilitated collaboration. A contractor who was skillful at organizing materials, facilitating meetings, motivating work group members, and responding to the needs of work group members was also essential. Because the work group volunteered its time to create the DIDIT, efforts were made to minimize the burden placed on its members. Minimizing this burden helped to maintain a core group of members who have actively participated for more than 3 years.

### Iterative development process

Development of both content and Web application took place incrementally and iteratively. Members of the work group reviewed the content in phases, allowing the content contractor to apply feedback to subsequent phases. Similarly, because an incremental process was conducted that involved analysis, design, and implementation at the same time, the contractors were able to make a demonstration model of the DIDIT during the early phases of development, which facilitated refinements to its content and design. Working with an actual tool triggered ideas among users for additional functions and alternative designs that may have been overlooked at the prototyping stage. A model also allowed us to obtain user input on database-driven features such as system searches.

## Implications for Public Health Practice

The ability to assess the status of the public's health in a timely, consistent, and accurate manner satisfies the first two of the 10 essential public health services as defined by the Institute of Medicine: 1) "monitor health status to identify community health problems" and 2) "diagnose and investigate health problems and health hazards in the community" (7).

The DIDIT represents an innovative approach to enhancing the capacity of state and federal agencies to perform public health surveillance. As one user has described, "The DIDIT offers a one-stop shop that is available 24 hours a day." It empowers users by providing them easy access to information that has been reviewed by DIDIT work group members for accuracy and content. In addition to providing a road map for development, this article highlights components that were critical to the successful development of the DIDIT. These components synergistically influenced the development process. Having adequate time, expertise, and commitment of resources, for example, would not have been sufficient for success without the clear communication and rapport among the project team members or buy-in and involvement of all stakeholders. Because these critical factors enhance one another, it is difficult to prioritize them. Other entities that wish to undertake a similar effort of systems development can use these requirements as guiding principles and customize them for their own needs and circumstances.

A major benefit of sharing these elements is to prevent other agencies from having to "reinvent the wheel" when they can draw directly on the experiences of the DIDIT team. While the technology is available to develop information technology solutions for addressing public health problems, it is vital to have effective processes and methods in place to successfully identify the needs of users and harness and customize appropriate technology to meet those needs.

## Figures and Tables

**Table 1:** Timetable for Development of the Diabetes Indicators and Data Sources Internet Tool (DIDIT), 2001–2005

**Dates**		**Key Steps**
August 2001–December 2001	Formed a work group composed of staff representing the Division of Diabetes Translation (DDT) at the Centers for Disease Control and Prevention (CDC) and eight Diabetes Prevention and Control Programs (DPCPs). Generated 55 diabetes indicators and drafted an outline of the objectives of a Web-based tool during concept development.	
	Conducted six focus groups in Atlanta, Ga, to assess needs and elicit input on draft vision statement from PDCP staff members.	
	Recruited additional work group members.	
	Revised the concept for tool based on DPCP feedback	
January 2002–December 2002	Selected 10 indicators to be piloted and finalized data fields that would describe indicators and their data sources, based on feedback from work group members.	
	Developed content for 10 pilot indicators with assistance from a contractor.	
	Presented revised concept of the DIDIT at annual conference for DDT.	
	Worked with contractor to develop a Web site that would house DIDIT information.	
	Selected an additional 28 indicators to be included in the DIDIT, using a two-round Delphi process.	
December 2002	Finalized development of demonstration Web site to be used for displaying and reviewing DIDIT content as it was developed.	
January 2003	Organized the first in-person meeting in Atlanta, Ga. The work group reviewed the demonstration Web site containing information on 10 pilot indicators and developed a process for reviewing the content for indicators and associated data sources.	
January 2003–March 2003	Revised content of 10 pilot indicators and completed content development of 28 additional indicators.	
	Uploaded indicators and data sources on the demonstration Web site.	
	Revised content based on work group feedback.	
	Obtained final approval of content.	
April 2003	Uploaded revised indicators and data sources on demonstration Web site.	
	Conducted DIDIT usability testing at DDT annual meeting.	
	Presented DIDIT demonstration model at DDT annual meeting.	
May–June 2003	Revised DIDIT content based on usability-test feedback.	
July 2003–August 2003	Pilot tested content, design, and functionality with nine DPCPs.	
August 2003	Revised content and design, based on pilot-test feedback and work group review.	
September 2003	Made available a live version of the DIDIT to all DPCPs and DDT staff.	
November 2003	Conducted a panel presentation describing and demonstrating the DIDIT at the annual American Public Health Association conference.	
December 2003	Presented the DIDIT at annual meeting of DPCP program directors.	
October 2003–April 2004	Provided training to CDC project development officers and DPCP staff.	
April 2004–January 2005	Presented the DIDIT at monthly DDT "All Hands" meeting.	
	Had users share DIDIT experiences at annual meeting of DPCP program directors.	
	Developed and released DIDIT glossary with 100 epidemiology and surveillance terms.	
	Developed and released a section in which users share DIDIT experiences.	
	Analyzed requirements for adding a section to provide users with resources on surveillance and epidemiology.	
January 2005–May 2005	Developed and conducted DIDIT evaluation with nine DPCPs.	
	Designed and developed resources section.	
	Finalized and tested protocol for updating DIDIT content.	

**Table 2:** Four Phases of the Software Development Life Cycle, Diabetes Indicators and Data Sources Internet Tool (DIDIT)

**Phases**	**Description**
1. Planning	Articulate objectives and scope of DIDIT systems; ensure technical feasibility.
2. Analysis	Define in detail the information system that will provide users with the benefits they desire.
3. Design	Focus on how the information system will function, including design of the application, database, user interface, and operating environment.
4. Implementation	Code, pilot test, and deploy the application; train users.
